# The Effect of *TAX-1* Gene of Human T-cell Leukemia Virus Type-1 on the Expression of CCR5 in K562 Cell Line

**Published:** 2019

**Authors:** Nasrin Haghnazari Sadaghiani, Lila Pirayeshfard, Afsaneh Aghaie, Zohreh Sharifi

**Affiliations:** Blood Transfusion Research Center, High Institute for Research and Education in Transfusion Medicine, Tehran, Iran

**Keywords:** CCR5, Human T-cell Leukemia Virus, Tax-1 protein

## Abstract

**Background:**

Tax-1 protein of Human T-cell Leukemia Virus type 1(HTLV-1) serves as a key transcriptional regulatory gene product and has a crucial role in transactivating genes of infected cells by employing their transcriptional factors. This modulation includes induction of genes which encode CC-chemokines and their receptors. In this study, a recombinant vector containing Tax-1 gene was made and tested for its ability to induce CCR5 (CC chemokine receptor 5) expression in K562 cell line.

**Methods:**

In order to perform this research, two blood samples of HTLV-1 positive were obtained from Urmia blood transfusion center. After DNA extraction, a complete sequence of *Tax-1* gene was amplified by specific primers. Recombinant vectors carrying *Tax-1* gene were synthesized and transformed into *Escherichia coli (E. coli).* After bacteria transformation, bacteria containing recombinant plasmid were selected and purified. Then, the recombinant shuttle vectors, *pCDNA3.1-TAX*, were transfected into the cell culture (K562 cell line). Expression of *CCR5* was measured after 72 *hr* by Syber Green Real-Time PCR method compared to control cell culture. Normalization was done with GAPDH as a standard gene.

**Results:**

Cloning of *Tax-1* gene in the vector, *pCDNA3.*1 was confirmed by colony PCR, restriction digestion, and sequencing methods. Expression of *Tax-1* and *CCR5* genes were confirmed by real time PCR and also, expression of *CCR5* gene showed an 8-fold increase in comparison to mock-treated controls (p<0.05).

**Conclusion:**

Our data suggested that recombinant Tax-1 may have the enhancing effect on CCR5 expression rate at mRNA levels in K562 cell line. Further studies are necessary to evaluate the effect of *pCDNA3.1-TAX* on cell surface CCR5 expression.

## Introduction

Human T-cell Leukemia Virus (HTLV-1) is the first discovered human retrovirus in 1980. It was isolated from a patient with cutaneous T-cell lymphoma 1. Worldwide distribution of infected individuals has suggested that 15 to 20 million people are infected with HTLV 2. Africa, southern Japan, Caribbean, Central and South America and the Middle East are the endemic areas for this infection. It is the etiological agent of Adult T-cell Leukemia/Lymphoma (ATL) and HTLV-1 Associated Myelopathy/Tropical Spastic Paraparesis (HAM/TSP) 3. However, only about 1–5% of infected patients are thought to develop cancer as a result of the infection with HTLV-I over their lifetimes 2. Infection is possible through sexual contact, transfusion of contaminated blood products, and from mother to child *via* breastfeeding 4. Entry of HTLV-1 into the CD4+ T-cells occurs through a ubiquitous cell surface transporter called Glucose Transporter 1 (GLUT1) 5, which is present on a wide range of cell types; however, they have a preferential tropism for CD4+ T-cells 6.

HTLV-1 belongs to retroviridea family and therefore, shares similar transmission modes with the other member, HIV-1, thus, both can be found in a host in endemic areas 7. Human retroviruses have the worldwide distribution but because of the lack of HTLV screening in most of the countries, its coinfection rate with HIV-1 has been underestimated. HTLV-1 and HIV-1 both infect CD4+ T-cells but have different outcomes on the same cell type. In spite of the long-lasting history of investigation of HTLV-1/HIV-1 coinfection, whether HTLV-1 can accelerate or attenuate HIV-1 infection has not been completely clarified as yet. Studies of clinical outcomes of the coinfection show conflicting results 8. What accounts for most of HTLV-1 influence on a host cell is Tax-1. Tax-1 is the oncogenic transactivator agent of HTLV-1 which causes several changes to an infected cell by modulating the transcription factors and pathways and shifting the mechanisms in favor of the virus replication 9. Furthermore, an important role was suggested for Tax-1 in a clinical and immunological manifestation of the HIV/HTLV coinfection 10. Tax-1 may have the capability to increase CCR5 expression in the Tax-1 transfected cell line.

Previous studies on this subject have had conflicting results of the effect of Tax-1 on CCR5 expression 11,12. In this study, the effect of Tax-1 on CCR5 expression in K562 cell line was investigated.

## Materials and Methods

### DNA purification and amplification

In order to isolate genomic DNA, two blood samples of HTLV-1 positive were obtained from Urmia blood transfusion center. DNA extraction was performed according to the method of extraction kit protocol (Yekta Tajhiz, Iran). Specific primers were designed using NCBI/Primer-Blast for the complete length of *Tax-1* gene in the NCBI nucleotide database as follows: A forward primer containing a restriction enzyme site of *HindIII* 5’ATAAGCTTATGGCCCACTT CCCAGGGTT3’ and reverse primer containing a restriction site of *EcoR1* 5’TAGAATTCTCATCATCTG CCTCTTTTTCGTT3’ with an amplicon size of 890 base pairs. The PCR amplification kit (TaKaRa bio kit, USA) is used in a total volume of 50 *μl* containing 25 *μl* of the master mix, 1 *μl* of isolated DNA and 1 *μl* of each primer (10 *μM*). PCR condition included a primary denaturation step of 5 *min* at 95°*C* followed by 35 cycles of 45 *s* at 95°*C*, 30 *s* at 58°*C*, and 45 *s* at 72°*C* and a final extension step at 72°*C* for 10 *min* using Palm cycler (Corbett, USA).

### Recombinant DNA construction

In order to construct the recombinant DNA, *Pc-DNA3.1* plasmid was used as the vector. The *PcDNA-3.1* plasmid was purified using a plasmid DNA miniprep system (Viogene, USA) from the bacterial culture containing the plasmids stored in glycerol stock according to the supplier's instructions. PCR products and the vectors were digested by *EcoR1* and *Hind III* producing sticky ends and ligation was done by T4 ligase overnight to yield in the recombinant *PcDNA3.1-Tax-1* plasmid. In order to make competent *Escherichia coli (E. coli)*, the *TG1* bacterial culture was treated with CaCl_2_ 100 *mM* solution. Transformation of the *TG1* culture with the recombinant vector was done with heat shock method. For confirmation of the recombinant *PcDNA3.1-Tax-1* plasmid, colony PCR was done to evaluate cloning. The accuracy of the recombinant *Pc-DNA3.1-Tax-1* plasmid was evaluated by DNA sequencing.

### Cell line transfection

K562 cell line was used as a myeloid cell line competent of producing CCR5 naturally. Cells were cultured in an RPMI medium with 10% FBS and 1% penicillin/streptomycin. Transfection was done with *Pc-DNA3.1-Tax-1* plasmids using XtremeGene Transfection kit (Roche, Germany) according to the manufacturer’s protocol. As a control, K562s were transfected with *PcDNA3.1* without the insert.

### Total RNA extraction and cDNA synthesis

Total RNA was extracted with RNA extraction kit (Yekta Tajhiz Azma, Iran) from cell culture after 72 *hr* of transfection. cDNAs were generated using cDNA synthesize kit (Invitrogen, USA) according to the manufacturer’s protocol.

### Quantitative real-time PCR

To indicate the level of the *Tax-1* and *CCR5* genes expression, quantitative real-time PCR was performed on cDNA samples using previously mentioned Tax-1 primers and CCR5 specific primers which are as follows: Forward primer: 5′CAAAAAGAAGGTCTTCA TTACACC3′ and reverse primer: 5′CCTGTGCCTCT TCTTCTCATTTCG3′ 13. In order to determine the optimal annealing temperature, thermal gradient series of PCR were performed and 58°*C* was determined as the proper annealing temperature for both *CCR5* and *Tax-1* genes. Quantitative real time PCR was performed using Syber Green qPCR master mix (Ampliqon, Denmark) with real-time PCR (Rotor-Gene, Q USA). PCR condition was a primary denaturation and enzyme activation step of 1 cycle of 15 *min* at 95°*C* which was followed by 40 cycles of amplification including a denaturation step of 45 *s* at 95°*C*, a 30 *s* at 58°*C* for annealing and a 45 *s* at 72°*C* for extension and a final cooling step of 30 *s* at 40°*C*. In order to control the specificity of the amplification products, a melting curve was generated with a temperature ramp from 60°*C* to 95°*C* at a rate of 0.01°*C/s*. Normalization was done with the internal control, *GAPDH* gene. Relative expression of each gene was calculated with the comparative ΔΔCt method 14, in which ΔCt=Ct (Tax-1 or CCR5)–Ct (GAPDH). The ΔΔCt method was used to find the difference between the mean value of the ΔCt of the transfected group and the mean value of the ΔCt of the control group (ΔΔCt = ΔCT_transfected_ - ΔCt_control_) for each analyzed molecule. Fold changes in the expression of target mRNA was calculated as 2^−ΔΔCt^. PCR efficiency for the gene of interest and for internal control must be similar which allows us to use the comparative Ct method. To determine the efficiency, tenfold dilution series were prepared for Tax-1 and CCR5 cDNAs and after real time PCR, a standard curve was established by plotting the logarithm of copy numbers in dilutions against the ΔCt values. The difference in PCR efficiency was determined by calculating the slope of the line.

## Results

### Amplification of the Tax-1 gene

**Figure 1.**
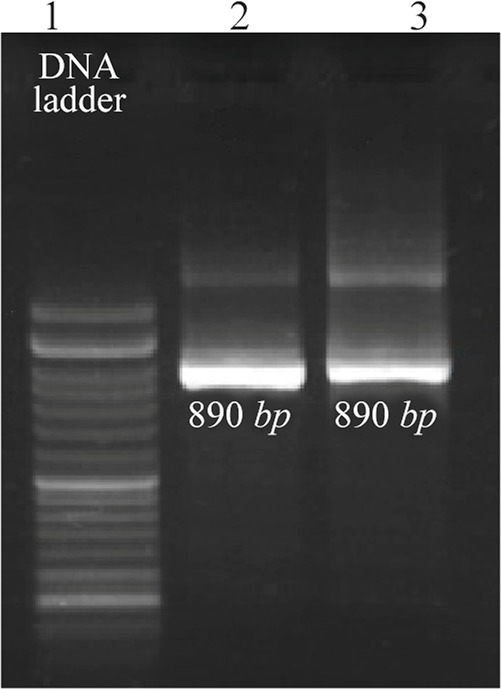
Agarose gel electrophoresis of PCR products of HTLV-1 positive samples. Line 1,100 *bp* DNA ladder. Lines 2 and 3, 890-*bp* PCR fragments of amplified *Tax-1* gene.

PCR products were electrophoresed on gel agarose and according to the 100 *bp* DNA ladder, an 890-*bp* PCR fragment was amplified (Figure 1).

### Confirmation of recombinant vector PcDNA3.1-Tax-1

**Figure 2.**
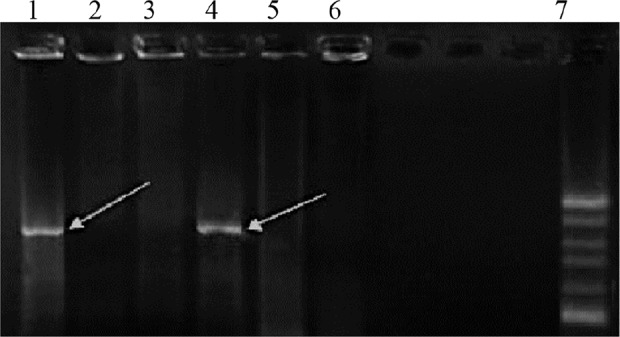
Agarose gel electrophoresis of colony PCR results of the selected clones containing recombinant plasmid PcDNA3.1-*Tax-1*. Lines 1 and 5, PCR products of the recombinant plasmid containing *Tax-1* genes with an 890-*bp* fragment. Lines 2, 3 and 6, PCR products of plasmid *PcDNA3.1* without the insert. Line 7,100 *bp* DNA ladder.

After colony PCR, products were electrophoresed on gel agarose and a fragment of about 890 base pairs was amplified using Tax-specific primers (Figure 2).

**Figure 3.**
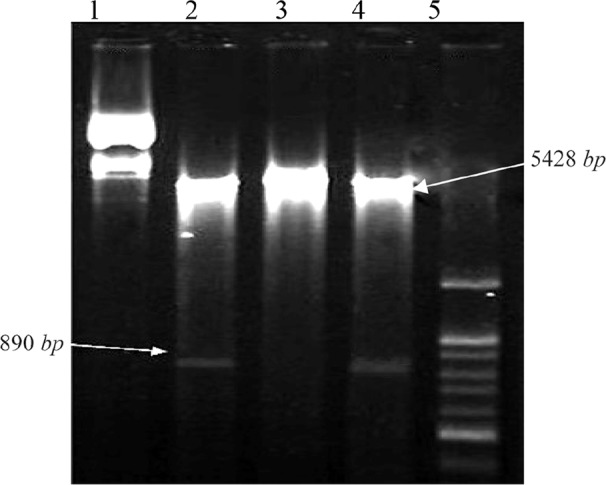
Agarose gel electrophoresis of double digestion of recombinant plasmid PcDNA3.1-*Tax-1* containing *Tax-1* gene. Lines 1 and 3, undigested recombinant plasmid *PcDNA3.1-Tax-1,* Lines 2 and 4, recombinant plasmid *PcDNA3.1*-*Tax-1* that was digested with *HindIII and EcoR1*enzymes with two fragments (PcDNA3.1, 5428-*bp*, and *Tax-1*, 890-bp). Line 5, 100 *bp* DNA ladder.

Also, the recombinant plasmid *PcDNA3.1-Tax-1* was digested with *EcoR1* and *Hind III* restriction enzymes that resulted in two fragments PcDNA3.1, 5428-*bp*, and *Tax-1*, 890-*bp* (Figure 3).

The result of DNA sequencing showed that this isolate had 99% similarity with *Tax-1* gene of the HTLV-1 virus with the accession number KF797883.1.

**Figure 4.**
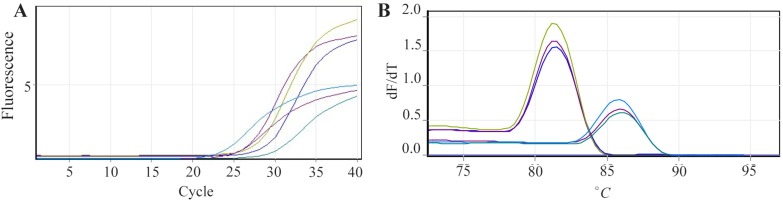
A) Amplification curves and; B) melt curve analysis of *CCR5* and *GAPDH* genes as target and control genes, respectively revealed melting peaks at 86°*C* for *CCR5* gene and 83°*C* for *GAPDH* gene and the representative amplification with gene specific primers.

Analyzing real time PCR results after 72 *hr* of transfection with recombinant plasmid *PcDNA3.1-Tax-1* indicated the expression of *Tax-1* gene in comparison to the control group (p<0.001). After confirmation of *Tax-1* gene expression in transfected cell line K562, samples were subjected to quantitative real time PCR to determine the effect of *Tax-1* on CCR5 expression (Figure 4). According to the relative expression results which were analyzed by a Relative Expression Software Tool (REST) and ΔΔCt method, there is an over-expression rate of 8 fold in CCR5 expression in comparison to mock-treated group (p<0.001).

## Discussion

HTLV-1 is one of the viruses with an incubation period of 10 to 40 years and in most of the patients, it has no manifestation during their lifetime, thus, patients in endemic areas with high risk behaviors are prone to coinfections with HIV-1 10. Although there has been a significant number of investigations on HTLV-1 infection throughout more than a quarter of a century, HTLV-1 significance in coinfection with HIV-1 has been underestimated due to lack of screening in most of the endemic areas 10. There have been reports of both enhancement and attenuation of HIV-1 replication in cases of coinfections with HTLV-1 15–20. Presence of HTLV-1 in the host can increase the chance of HIV-1 to get into the cells and therefore increasing the chance of HIV-1 infection. HTLV-1 has a transactivator agent named Tax-1, which can cause a series of transformations into the host cell. One of the modifications which is of importance in HTLV-1/HIV-1 coinfection is the modulation of CCR5 expression rate which is one of the co-receptors helping HIV-1’s entry into the cells 21.

There have been a few publications about the effect of *Tax-1* gene on CCR5 expression rate. In a study, it was shown that a higher percentage of classical monocytes subset (CD14^+^ and CD16^−^) expressed chemokine receptor CCR5 among infected individuals with HT-LV-1 in comparison to uninfected individuals 22.

In another study, when healthy PBMC cultures were treated with different doses of Tax-1 protein, there was an increase in CC-chemokines such as CCL3, CCL4, and CCL5 and a reduction in the percentage of CCR5-positive cells compared to mock-treated lymphocytes which were evaluated by flow cytometry 11.

Studies showed that Tax1 protein is a potent trans-activator that regulates the expression of several cellular genes involved in cellular activation, proliferation, and transformation. For example, Tax-1 has been shown to be a relatively weak activator of the CXCR4 promoter 23. Also, in Twizere *et al*’s study, it was reported that Tax-1 would induce the expression of several proteins, including cytokines and chemokines after infection of T-cell by HTLV-1. But Tax-1 expression in primary T lymphocytes did not result in altered cell surface expression of CXCR4 in comparison with control cells and they demonstrated that Tax-1 could modify the binding properties of the CXCR4 chemokine receptor and induce CXCR4 receptor activation 24.

A possible explanation for the discrepancy of this study with previous studies can be due to evaluation of CCR5 expression rate by real time PCR which is an accurate and sensitive method for quantifying gene transcription. In this research, the expression of CCR5 gene was calculated at a transcriptional level without measuring cell surface CCR5 expression and the possible interaction with others proteins such as CC-chemokines or quantifying CC-chemokines expression.

The CCR5 expression is regulated at three levels: (a) genetic factors; (b) factors involved in activation, signaling, and trafficking of the receptors and; (c) environmental or other triggers 25. The CCR5 expression is mainly affected by the binding of its ligand; however, studying the direct effect of *Tax-1* protein as a trans-activator on the CCR5 promoter and expression rate can be useful. It is reported that CCR5 expression rate increased autocrine effect of MIP1β expression which was induced by mitogens in a *tax-1* transfected cell line 12. Furthermore, the rate of expression of the recombinant *PcDNA3.1-Tax-1* plasmid and required time for its uptake by K562 cell line should be considered.

## Conclusion

Our data suggested that recombinant *Tax-1* may have the enhancing effect on CCR5 expression rate at mRNA levels in K562 cell line. Further studies are necessary to evaluate the effect of *pCDNA3.1-TAX* on cell surface CCR5 expression.
